# Optimizing diabetes classification with a machine learning-based framework

**DOI:** 10.1186/s12859-023-05467-x

**Published:** 2023-11-13

**Authors:** Xin Feng, Yihuai Cai, Ruihao Xin

**Affiliations:** 1 School of Science, Jilin Institute of Chemical Technology, Jilin, 130000 People’s Republic of China; 2 State Key Laboratory of Inorganic Synthesis and Preparative Chemistry, College of Chemistry, Jilin University, Changchun, 130012 People’s Republic of China; 3 Department of Epidemiology and Biostatistics, School of Public Health, Jilin University, Changchun, 130012 People’s Republic of China; 4 College of Information and Control Engineering, Jilin Institute of Chemical Technology, Jilin, 130000 People’s Republic of China; 5 College of Computer Science and Technology, and Key Laboratory of Symbolic Computation and Knowledge Engineering of Ministry of Education, Jilin University, Changchun, 130012 People’s Republic of China

**Keywords:** Diabetes diagnoses, Machine learning, GAN

## Abstract

**Background:**

Diabetes is a metabolic disorder usually caused by insufficient secretion of insulin from the pancreas or insensitivity of cells to insulin, resulting in long-term elevated blood sugar levels in patients. Patients usually present with frequent urination, thirst, and hunger. If left untreated, it can lead to various complications that can affect essential organs and even endanger life. Therefore, developing an intelligent diagnosis framework for diabetes is necessary.

**Result:**

This paper proposes a machine learning-based diabetes classification framework machine learning optimized GAN. The framework encompasses several methodological approaches to address the diverse challenges encountered during the analysis. These approaches encompass the implementation of the mean and median joint filling method for handling missing values, the application of the cap method for outlier processing, and the utilization of SMOTEENN to mitigate sample imbalance. Additionally, the framework incorporates the employment of the proposed Diabetes Classification Model based on Generative Adversarial Network and employs logistic regression for detailed feature analysis. The effectiveness of the framework is evaluated using both the PIMA dataset and the diabetes dataset obtained from the GEO database. The experimental findings showcase our model achieved exceptional results, including a binary classification accuracy of 96.27%, tertiary classification accuracy of 99.31%, precision and f1 score of 0.9698, recall of 0.9698, and an AUC of 0.9702.

**Conclusion:**

The experimental results show that the framework proposed in this paper can accurately classify diabetes and provide new ideas for intelligent diagnosis of diabetes.

## Introduction

Diabetes is a chronic disease resulting from insufficient insulin production by the pancreas or ineffective insulin use by the body [1]. Without enough insulin, glucose absorption is hindered, resulting in increased blood glucose levels that can damage various organs over time. While diabetes cannot be cured, it can be managed through careful diet, physical activity, medication, and regular screening for complications. Failure to treat diabetes can result in severe complications such as cardiovascular disease, diabetic ketoacidosis, chronic kidney disease, and foot ulcers, among others [2]. Shockingly, the number of people with diabetes has risen from 108 million in 1980 to 422 million in 2014, with an estimated 700 million people projected to have diabetes by 2045 [3]. Thus, developing an intelligent diagnostic framework for diabetes is crucial, given the disease's significant impact.

The disease has three types: type 1 diabetes, type 2 diabetes, and gestational diabetes [4]. Type 2 diabetes, affecting over 95% of diabetic patients, results from the body's inability to use insulin efficiently, typically caused by being overweight and lacking physical activity. In contrast, type 1 diabetes results from insufficient insulin secretion, requiring insulin injections, and its cause remains unknown. Polyuria, thirst, hunger, weight loss, and vision loss are specific symptoms of type 1 diabetes. Finally, gestational diabetes, a hyperglycemic condition, occurs during pregnancy when blood glucose levels are higher than usual average but not high enough for a diabetes diagnosis.

In recent years, machine learning has received increasing attention in medicine, particularly for intelligent disease diagnosis. Consequently, machine learning techniques have been widely applied to the intelligent diagnosis of diabetes [5]. By analyzing and mining data from diabetic patients, machine learning models can help with early diagnosis, classification, prediction, and treatment planning. With the promise of improving diabetes management and treatment, researchers are exploring the application of machine learning technology in diabetes diagnosis.

However, despite recent advances, several challenges remain. Data acquisition and processing challenges plague many disease diagnosis areas, including small, unbalanced, or low-quality data, which can impact algorithm performance.

The PIMA dataset presents several challenges of complexity, including class imbalance, a significant number of missing values, and low data quality. Previous studies utilizing simple machine learning techniques have yielded subpar model performance and unsatisfactory results on the PIMA dataset. Similarly, attempts by researchers to employ complex deep learning models have not proven effective in addressing these challenges, despite their intricacy.

To address these challenges, this paper proposes a machine learning-based framework.

The major contributions of this study summarized as follows: Firstly, a novel imputation technique combining mean and median values is employed to address missing data. This imputation method not only fills in the missing values but also helps in making the data distribution more normal. And outliers were effectively handled using a capping method. These strategies ensured the integrity and accuracy of the dataset, enhancing its reliability for subsequent analyses.

Secondly, the SMOTEENN algorithm was utilized to mitigate the issue of data imbalance. By integrating the Synthetic Minority Over-sampling Technique (SMOTE) with the Edited Nearest Neighbors (ENN) approach, the SMOTEENN method successfully balanced the representation of minority and majority classes. This alleviated the inherent bias arising from imbalanced data and subsequently enhanced the performance of the classification model.

Furthermore, the research introduces the DCSGAN model, which has shown promising results in achieving high accuracy in diabetes diagnosis. The DCSGAN leverages the power of generative adversarial networks to continuously generate synthetic samples during training. This augmentation of the training process enhances the discriminative capability of the model, enabling it to capture intricate patterns and features that are essential for accurate diabetes classification. The high accuracy achieved by the DCSGAN model contributes to the reliability and effectiveness of diabetes diagnosis using machine learning technology. The DCSGAN model proposed in our study demonstrates outstanding performance not only in the PIMA dataset but also surpasses the performance of other models in the GEO database. This highlights the superiority and effectiveness of our proposed model in accurately predicting and classifying diabetes cases in diverse datasets.

**Fig. 1:**
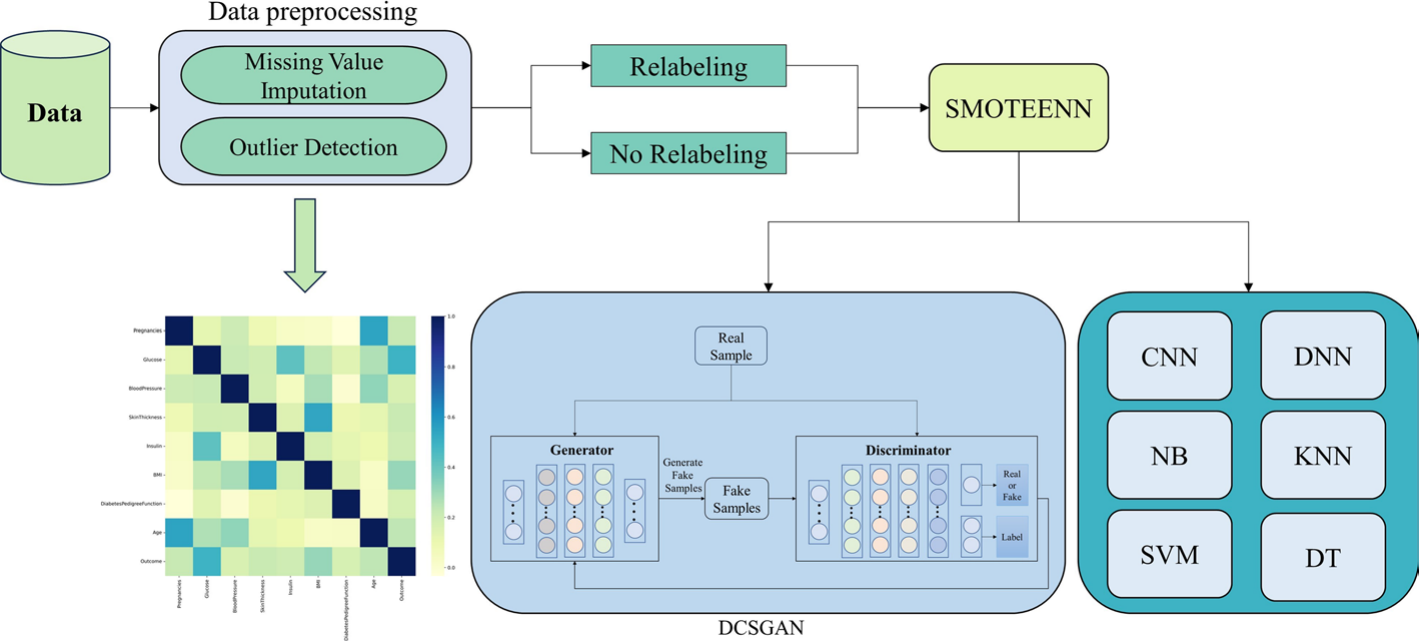
The MOG framework

The paper is organized as follows: The Related Work section discusses current research in diabetes classification and the challenges that scholars face. The Materials and Methods section describes the dataset, data preprocessing techniques, and the diabetes classification model (DCSGAN). The Results section presents the framework's results, including comparisons to other classifiers, different classification tasks, and results on different datasets. Figure 1 shows the flow chart of our MOG framework.

## Related work

In recent years, the advancement of computer technology has led to the flourishing of machine learning. As a result, an increasing number of scholars are applying machine learning techniques to improve the diagnosis and treatment of diabetes.

Several studies have employed traditional machine learning classifiers for diabetes prediction and classification. Saxena et al. [6] used the K-nearest neighbor algorithm and achieved a 79.8% accuracy rate, whereas Krishnamoorthi et al. [7] proposed logistic regression for data classification. Butt et al. [8] conducted an extensive study with three classifiers: random forest, multilayer perceptron, and logistic regression. Their study demonstrated the superior performance of the multilayer perceptron classifier, achieving an accuracy of 86.06%. Another study by Zou et al. [9] also implemented decision trees, random forests, and neural networks for diabetes prediction and found that random forest had the highest accuracy of 80.84% when all features were employed.

Maniruzzaman et al. [10] applied an ensemble of ten different classifiers, and their highest accuracy rate was a significant 92.26%. Similarly, Maniruzzaman et al. [11] utilized Gaussian Process-based classification technology, with an accuracy rate of 81.97%. Joshi and Dhakal [12] used logistic regression models and decision tree algorithms, achieving a prediction accuracy of 78.26%.

Deep learning has been increasingly employed in recent years due to its superior capacity to handle complex data. Garcia-Ordas et al. [13] utilized a variational self-encoder for data augmentation and a sparse self-encoder for feature augmentation. Their joint training of a convolutional neural network and a sparse self-encoder achieved an impressive 92.31% accuracy. Hasan et al. [14] used ensemble classifiers such as AdaBoost and Gradient Boost, and Bukhari et al. [15] proposed an improved ANN model, both without any data preprocessing. Rahman et al. [16] developed a novel diabetes classification model based on Convolutional Long Short-term Memory (Conv-LSTM), with the highest accuracy of 91.38%. And not only that Rehman et al. [17] proposed a deep extreme learning machine (DELM) prediction model, which achieved a reliability and accuracy rate of 92.8%.

Several studies have emphasized the importance of data preprocessing techniques and feature selection in improving prediction accuracy. A study by [18] tackled the issue of missing data by filling in the mean of each column. They trained six different models, with the XGBoost model achieving the highest accuracy rate of 77.54%. Hayashi and Yukita [19] proposed to use a rule extraction algorithm Re-RX with J48 graft, combined with a sampling selection technique to achieve an accuracy of 83.83%. Alneamy et al. [20] proposed an algorithm based on The Teaching Learning-Based Optimization (TLBO) algorithm and a new classification technique. Chang et al. [21] employed three interpretable supervised machine learning models and concluded that the Naive Bayes model is suitable for more refined binary feature selection.

Ejiyi et al. [22] proposed robust frameworks for predictive diabetes diagnosis using limited medical data. They identified glucose, age, and BMI as the most important features for prediction using SHAP, with XGBoost and Adaboost performing best. Lastly, Johora et al. [23] proposed a method involving image preprocessing and feature extraction for diabetic retinopathy detection. The results demonstrated superior performance, even for the noisy dataset. Jadhav et al. [24] automated the detection of diabetic retinopathy by analyzing retinal abnormalities, achieving significantly higher accuracy.

Studies such as Alam Miah et al. [25] focused on identifying risk factors for Type 2 diabetes. They collected data from patients and categorized the risk factors into socioeconomic conditions, habits, family history, and hard diseases. The study revealed significant factors affecting the quality of life in Type 2 diabetes patients. The relevant literature discussed above has been organized and presented in Table 1 for easy reference and comparison.

**Table 1:** Classification accuracy of different methods with literature

Authors	Preprocessing techniques	Models	Accuracy (%)
Saxena et al. [6]	Feature selection outlier rejection missing value padding	K-nearest neighbor, Random forest	79.80
Krishnamoorthi et al. [7]	Missing value processing, outlier removal, normalization	Logistic regression	83.00
Butt et al. [8]	Various classifiers and models	Random forest, multilayer perceptron, LSTM	86
Garcia-Ordas et al. [13]	Variational self-encoder, sparse self-encoder	Convolutional neural network, sparse self-encoder	92.31
Bukhari et al. [15]	No data preprocessing	Artificial back propagation proportional conjugate gradient neural network (ABP-SCGNN)	93
Gnanadass [18]	Missing data filling (mean)	Naive Bayes, linear regression, random forest, AdaBoost gradient boosting machine, extreme gradient boosting	78
Maniruzzaman et al. [10]	Missing data and outlier handling feature extraction and optimization	Ten different classifiers	92.26
Zou et al. [9]	Dimensionality reduction (PCA, mRMR)	Decision trees, random forests, neural networks	80.84
Hayashi and Yukita [19]	Rule extraction algorithm, sampling selection technique	J48 graft, rule extraction	83.83
Alneamy et al. [20]	TLBO algorithm, hybrid fuzzy wavelet neural network	Functional fuzzy wavelet neural network (FFWNN)	88.67
Maniruzzaman et al. [11]	Gaussian Process-based classification, three kernel functions	Gaussian process, LDA, QDA, NB	81.97
Joshi and Dhakal [12]	Logistic regression, decision tree	Logistic regression, decision tree	78.26
Ejiyi et al. [22]	Data augmentation, attribute analysis missing data imputations	XGBoost, adaboost	94.67
Rahman et al. [16]	Convolutional long short-term memory	Conv-LSTM, CNN, T-LSTM, CNN-LSTM	91.38
Rehman et al. [17]	Handling Miss values, moving average normalization	Deep extreme learning machine (DELM)	92.80

While the aforementioned investigations have made notable contributions to the domain of diabetes prediction and classification, it is imperative to discern certain potential drawbacks or limitations. These limitations encompass:

- *Data Preprocessing* Several studies have employed elementary data preprocessing techniques or complex algorithms which may have substantially modified the underlying data distribution. In contrast, our study employed the mean and median joint filling method to address missing values and implemented mean and median joint filling method to handle outliers. These meticulously chosen approaches aimed to uphold the data's integrity while facilitating a transition towards a more Gaussian or normal distribution.
- *Data Imbalance Handling* It is noteworthy that a limited number of studies have specifically addressed the issue of data imbalance within the PIMA dataset, while the majority of researchers have overlooked this aspect altogether. In the present study, we have employed the SMOTEENN algorithm as a means to effectively mitigate the problem of imbalanced samples in the PIMA dataset.
- *Classification Model* The majority of the existing research in the field has predominantly relied on simple machine learning algorithms, which may not yield satisfactory levels of accuracy and reliability. Conversely, a subset of researchers has delved into the utilization of more sophisticated models. However, there remains ample room for advancement in terms of enhancing model performance. Additionally, the generalizability of these models may be subject to question, as their validation has been limited to specific datasets and their performance on other datasets remains unexplored. Consequently, there is a need for further research to validate and assess the robustness of these models across diverse datasets to establish their applicability and effectiveness in real-world scenarios.

In response to the limitations observed in the aforementioned studies, we have devised the MOG framework as a means to overcome these deficiencies. Within this framework, we have employed an exhaustive and precise array of data preprocessing techniques, and introduced the DCSGAN model to augment the accuracy of diabetes classification.

## Materials and methods

### Dataset

This study utilized the PIMA Indian Diabetes Dataset, a publicly accessible dataset collected and compiled by the National Institute of Diabetes and Digestive and Kidney Diseases (NIDDK). The database comprises data from 768 patients, including 268 individuals with diabetes and 500 individuals without. For each patient, eight physiological indicators were recorded, namely Pregnancies, Glucose, Blood Pressure, Skin Thickness, Insulin, BMI, Diabetes Pedigree Function, and Age. These parameters were utilized to predict the presence of diabetes in each individual. Table 2 provides a detailed description of each characteristic.

**Table 2:** Description of PIMA dataset

S/N	Features	Description
1	Pregnancies	Number of times pregnant
2	Glucose	Plasma glucose concentration 2 h in an oral glucose tolerance test
3	Blood pressure	Diastolic blood pressure (mm Hg)
4	Skin thickness	Triceps skin fold thickness (mm)
5	Insulin	2-Hour serum insulin (mu U/ml)
6	BMI	Body mass index (weight in kg/(height in m)^2)
7	Diabetes pedigree function	Diabetes pedigree function
8	Age	Age (years)

The Gene Expression Omnibus (GEO) is an open-access repository designed for the preservation and dissemination of gene expression data. Maintained by the National Center for Biotechnology Information (NCBI), this vast database houses diverse genomics datasets, encompassing gene microarray data, RNA-Seq data, and miRNA data, among others. To evaluate the generalizability of our model, we selected 13 diabetes-related datasets from the GPL570 platform of GEO. A comprehensive overview of the chosen datasets is provided in Table 3.

**Table 3:** Description of GEO dataset

ID	Dataset	Samples	Features
1	GSE76894	103	29,530
2	GSE76895	103	29,612
3	GSE23343	17	54,613
4	GSE161355	33	54,675
5	GSE71416	20	54,675
6	GSE55650	23	54,613
7	GSE55100	44	54,675
8	GSE55098	22	54,675
9	GSE55099	22	847
10	GSE15932	32	54,675
11	GSE19420	42	54,675
12	GSE66738	14	45,101
13	GSE25462	50	54,675

### Data preprocessing

#### Missing value imputation

**Fig. 2:**
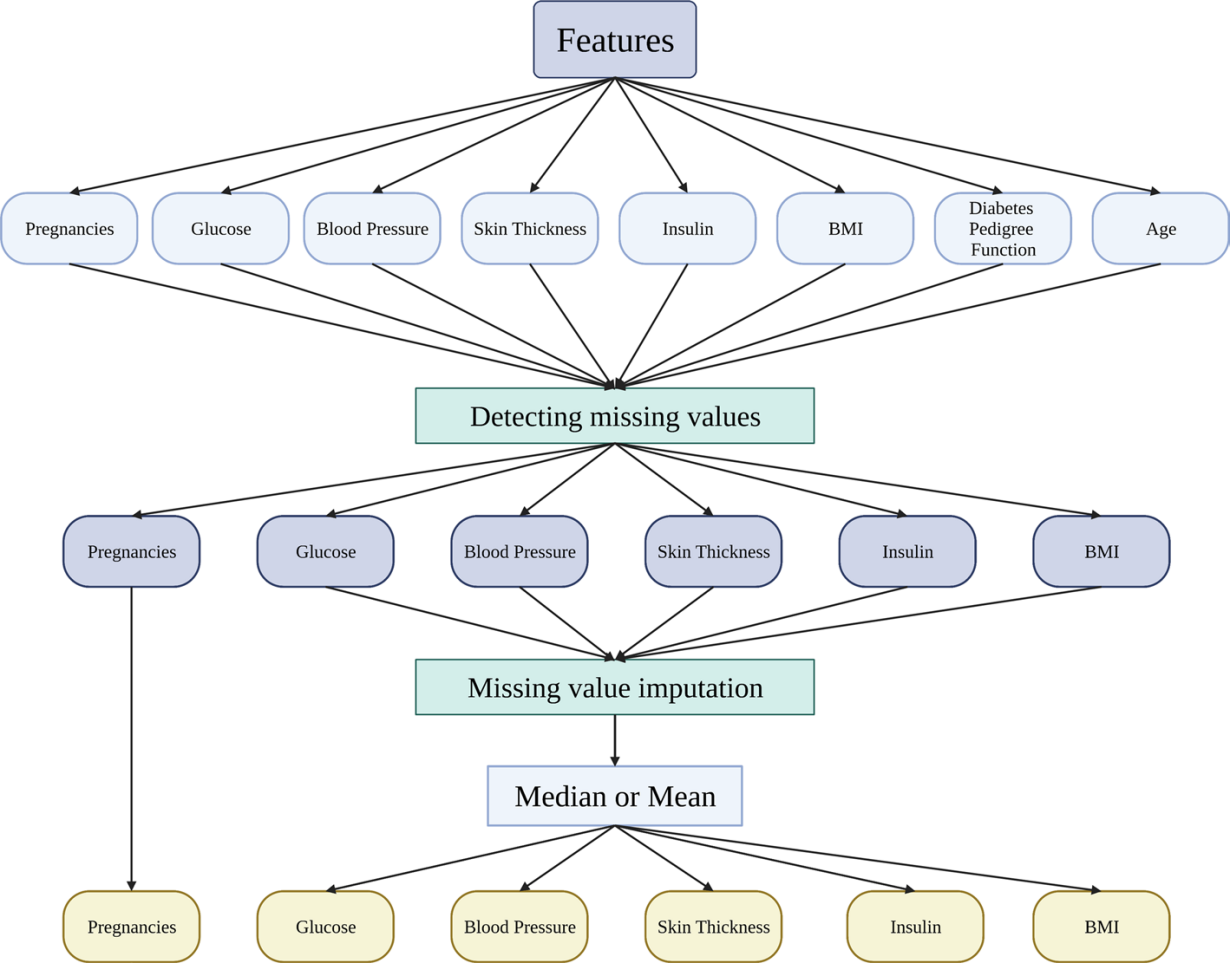
Mean and median joint filling method

Missing values are a common challenge in data analysis and machine learning, which occur when certain variables or attributes lack values during data collection or processing [26]. Missing values can cause problems such as reduced sample size, information loss, and biased analysis results, potentially compromising the accuracy and reliability of data analysis and models. Therefore, this paper addresses this issue by performing missing value processing on the data. Specifically, we identified 0 values in Glucose, Blood Pressure, Skin Thickness, Insulin, and BMI features in the PIMA dataset, which do not align with typical human indices, and treated them as missing values. To fill in the missing values, we utilized a combined mean and median filling approach to ensure the data distribution remains consistent with the normal distribution while minimizing any potential data bias. Figure 2 illustrates the schematic representation of our proposed technique for handling missing values.

#### Outlier detection

**Fig. 3:**
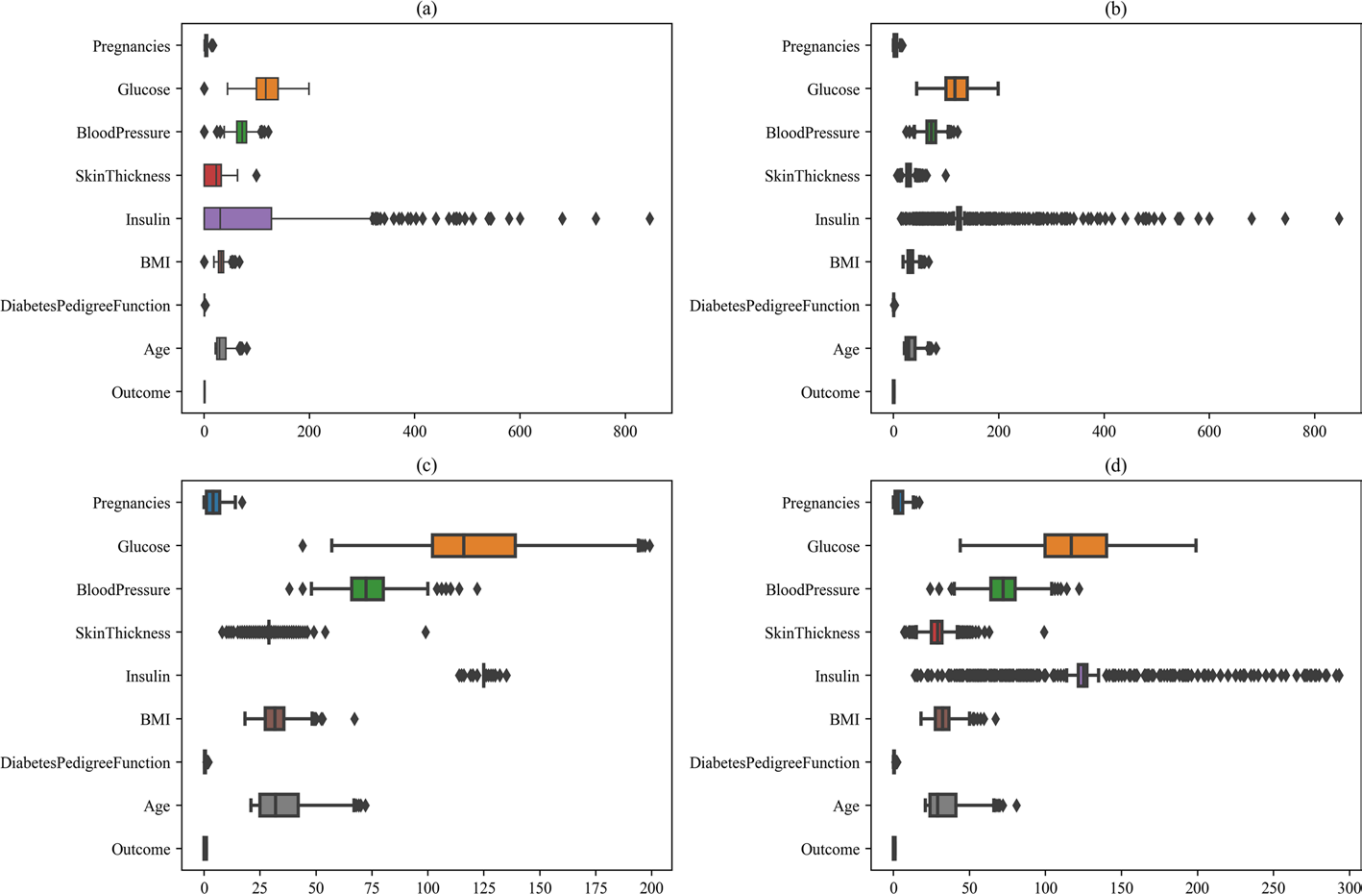
**a**–**d** Box plots of outliers of features. **a** Box plot of raw data. **b** Box plot of data after filling in missing values. **c** Box plot of data after removal of insulin outliers. **d** Box plot of the data processed by the capping method

Outliers, which are values in a dataset that are significantly different from other data values and can adversely affect the distribution, relationships, and statistical analysis of the data [27], must be identified and processed to obtain accurate data analysis results. As shown in Fig. 3, the box plot of the data indicates that the Insulin feature in the original data contains a large number of outliers that persist even after filling in missing values. As such, outlier processing is required for this feature to improve the quality of the analysis.

The direct removal of outliers is a frequently employed technique to handle these values. The basic principle involves the elimination of the outlier data points from the dataset. This method is straightforward and practical, particularly in cases where the dataset is large and the number of outliers is minimal. However, it is not without its drawbacks. Firstly, if the size of the sample after outlier removal is too small, the analysis results may be unreliable. Secondly, the deletion of outliers may result in a loss of valuable information, which can compromise the thoroughness and precision of the data analysis. Finally, as outliers in the data are often a mix of real occurrences and noise, the removal of outliers may incorrectly assess genuine data, consequently impacting the data analysis outcomes.

The capping method is a data preprocessing technique that mitigates the effect of outliers by transforming extreme values into more reasonable ones. This is achieved by computing the quartiles $Q1$, $Q2$, and $Q3$ of the data, where $Q1$ denotes the value below which 25% of the data lies and $Q3$ denotes the value above which 75% of the data lies. The formula for calculating IQR is shown in Eq. 1, the formula for upper and lower is shown in Eqs. 2 and 3.

1 $$\begin{array}{*{20}c} {IQR = Q3 - Q1} \\ \end{array}$$

2 $$\begin{array}{*{20}c} {upper = Q3 + 1.5 \times IQR} \\ \end{array}$$

3 $$\begin{array}{*{20}c} {lower = Q1 - 1.5 \times IQR} \\ \end{array}$$

The capping method offers several advantages for dealing with outliers. Firstly, it is straightforward and does not require any assumptions about the data distribution. Secondly, it can prevent outliers from exerting a significant impact on data conclusions. Additionally, compared to directly removing outliers, the capping method can prevent excessive reduction in the sample size, thereby preserving the integrity of the data for subsequent analytical processing.

#### Relabeling based on glucose

In machine learning, relabeling involves updating the labeling or classification of samples in a dataset to improve model performance and accuracy by correcting mislabeled or inaccurate labels. Table 3 demonstrates the diagnostic criteria for diabetes. Olisah et al. [28] relabeled the PIMA dataset by labeling samples with Glucose greater than 125 as diabetes, those with Glucose greater than 99 and less than or equal to 125 as prediabetes, and the remaining samples as normal based on Fasting Plasma Glucose show in Table 4. This transformed the PIMA dataset from a dichotomous to a trichotomous task. In this paper, to explore the model's generalization, the PIMA dataset is also transformed into a triple classification task based on Glucose.

**Table 4:** Criteria of diagnosing diabetes

Diagnosis	A1C	Fasting plasma glucose	Oral glucose tolerance test	Random plasma glucose test
Normal	below 5.7%	99 mg/dL or below	139 mg/dL or below	N/A
Prediabetes	5.7–6.4%	100 to 125 mg/dL	140 to 199 mg/dL	N/A
Diabetes	6.5% or above	126 mg/dL or above	200 mg/dL or above	200 mg/dL or above

#### Data imbalance handling with SMOTEENN

Data imbalance, characterized by significant variations in sample sizes among different categories in a classification problem, poses several challenges for machine learning models. Primarily, it introduces bias into the decision boundaries, leading to decreased accuracy when classifying minority categories. Additionally, model evaluation is distorted as the performance of minority categories is overshadowed by dominant categories in overall metrics like accuracy. The generalization ability of models is compromised, impairing their capacity to accurately classify unseen samples. Moreover, data imbalance can lead to erroneous predictions, where minority instances are misclassified as majority categories and vice versa. To address these challenges, various techniques such as resampling, ensemble methods, and cost-sensitive learning are employed to rebalance the data distribution and enhance the performance of models.

SMOTEENN is a hybrid sampling technique commonly employed to address the challenge of data imbalance encountered in classification tasks. This approach integrates the Synthetic Minority Over-sampling Technique (SMOTE) and Edited Nearest Neighbors (ENN) methodologies to rebalance the dataset and enhance the performance of machine learning models.

The SMOTEENN procedure consists of two main steps. Firstly, the SMOTE algorithm is applied, which generates synthetic samples for the minority class by interpolating feature vectors between neighboring instances. This augmentation process aims to improve the representation of the minority class and alleviate the class distribution imbalance.

Subsequently, the ENN technique is employed on the combined dataset, involving the identification and removal of noisy and ambiguous instances from both the majority and minority classes. ENN focuses on eliminating samples that are misclassified by their nearest neighbors, thereby enhancing the overall quality and separability of the dataset.

By leveraging the strengths of SMOTE and ENN, SMOTEENN effectively tackles the challenges posed by data imbalance. It addresses the underrepresentation of the minority class by synthesizing new samples, while simultaneously reducing noise and enhancing the discrimination between classes through the ENN step.

### Correlation analysis

#### Pearson correlation coefficient

Pearson's correlation coefficient is a valuable tool for assessing the strength of the linear relationship between two variables. This statistic ranges from -1 to 1, with 0 indicating no correlation, 1 indicating a perfectly positive correlation, and -1 indicating a perfectly negative correlation. The calculation formula for Pearson's correlation coefficient involves dividing the covariance of the two variables by the product of their standard deviations and is expressed as Eq. 4:

4 $$\begin{array}{*{20}c} {\rho_{X,Y} = \frac{{{\text{cov}} \left( {X,Y} \right)}}{{\sigma_{X} \sigma_{Y} }} = \frac{{E\left[ {\left( {X - \mu_{X} } \right)\left( {Y - \mu_{Y} } \right)} \right]}}{{\sigma_{X} \sigma_{Y} }}} \\ \end{array}$$

The variables ${\sigma }_{X},{\sigma }_{Y}$ represent the sample standard deviation, while ${\mu }_{X},{\mu }_{Y}$ represent the sample mean in the calculation formula.

#### Logistic regression

Logistic regression is a popular machine learning algorithm for binary classification, which is often used to analyze the impact of one or more independent variables on the dependent variable. The formula for logistic regression is as Eq. 5:

5 $$\begin{array}{*{20}c} {f\left( x \right) = \frac{L}{{1 + e^{{ - k\left( {x - x_{0} } \right)}} }}} \\ \end{array}$$

The logistic regression model employs a formula that includes an upper exact bound $L$ and a logistic growth rate $k$. Although it is commonly used as a classification algorithm, it is also valuable for correlation analysis, enabling the determination of whether two variables are correlated. One key advantage of using logistic regression for correlation analysis is its ability to accurately quantify the correlation between two variables, as well as analyze the correlation between multiple independent variables.

### DCSGAN: optimized for diabetes classification

Generative Adversarial Networks [29] consist of two neural networks, namely the Generator and the Discriminator. The Generator is responsible for generating synthetic samples by learning from the real data, with the objective of deceiving the Discriminator. On the other hand, the Discriminator learns to differentiate between real and generated samples.

The training process of GANs involves the Generator maximizing the probability of the Discriminator making mistakes, while the Discriminator aims to minimize the probability of misclassification. The core idea behind GAN is to generate synthetic data that closely resemble the distribution of real data. In the traditional GAN framework, the Discriminator outputs two categories (true and false) through a softmax output layer, indicating the likelihood of a sample belonging to the Generator's distribution. The objective function of GAN is shown in Eq. 6:

6 $$\begin{array}{*{20}c} {\min _{G} \max _{D} V\left( {D,G} \right) = E\left[ {\log D\left( x \right)} \right] + E\left[ {\log \left( {1 - D\left( {G\left( z \right)} \right)} \right)} \right]} \\ \end{array}$$

where $E$ represents the expectation of the training data distribution, $x$ denotes the genuine sample, and $z$ denotes the input noise distribution, the objective function can be decomposed into two terms. The first term, $E[log(D(x))]$, incentivizes the discriminator to accurately classify the genuine samples as 1. Conversely, the second term, $E[log(1 - D(G(z)))]$, encourages the generator to generate synthetic samples capable of misleading the discriminator.

In a modified version called SGAN [30], the Discriminator has N + 1 output units, including additional labels that can be utilized for classification tasks.

**Fig. 4:**
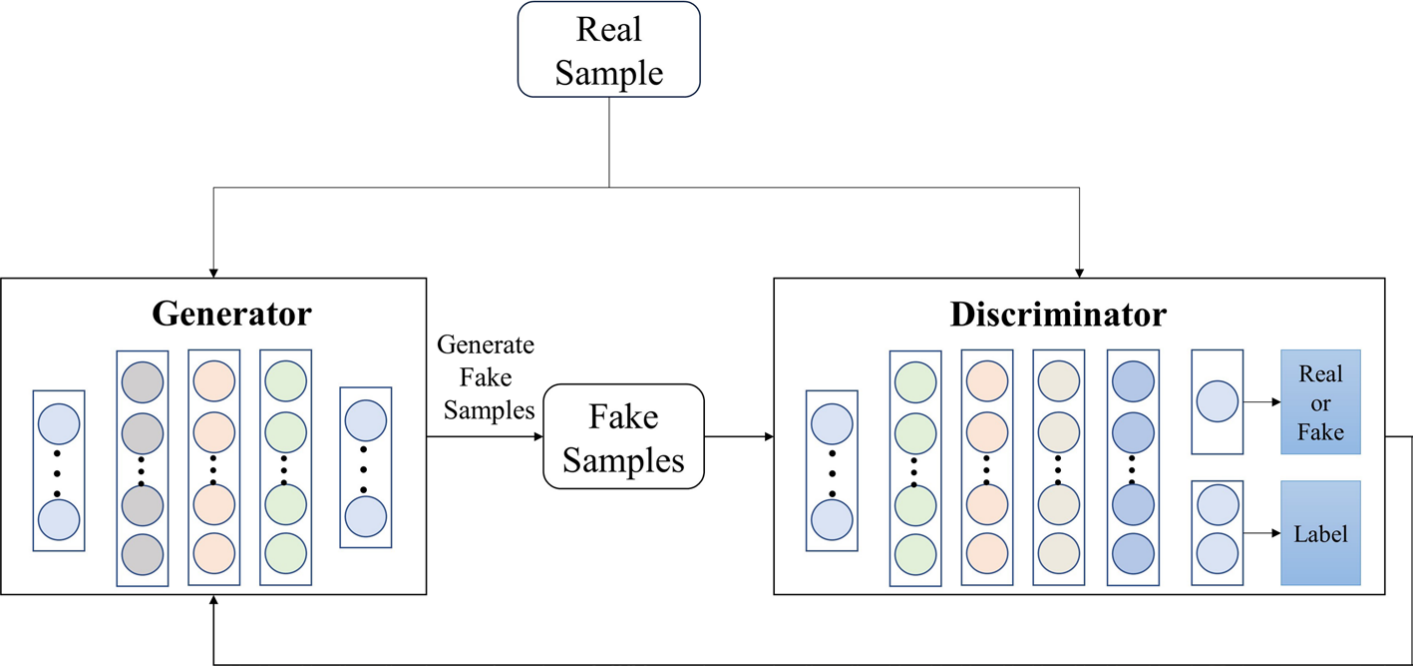
The architecture of DCSGAN

In this paper, we propose the DCSGAN, which leverages the principles of adversarial neural networks for diabetes classification. Figure 4 illustrates the architectural design of our proposed model. Initially, the Generator learns from the real data and generates synthetic data to deceive the Discriminator, which in turn attempts to distinguish between real and fabricated data through continuous training. As the Generator's performance improves and reaches a certain threshold, the synthetic data generated becomes indistinguishable from the authentic data. The objective function of DCSGAN is shown in Eq. 7:

7 $$\begin{array}{*{20}c} {\min _{G} \max _{D} \max _{C} V\left( {D,G,C} \right) = E\left[ {\log D\left( x \right)} \right] + E\left[ {\log \left( {1 - D\left( {G\left( z \right)} \right)} \right)} \right] + \lambda L_{C} \left( {C\left( x \right),y} \right)} \\ \end{array}$$

where ${L}_{C}$ denotes the loss function of classifier C, $C(x)$ denotes the classification result of the classifier on the real sample, and $y$ denotes the label of the real sample. The formula for ${L}_{C}$ is shown in Eq. 8:

8 $$\begin{array}{*{20}c} {L_{C} = - \frac{1}{N} \sum \left[ {\sum \left( {y \log \left( p \right) } \right)} \right]} \\ \end{array}$$

Through the optimization of the objective function $V\left(D,G,C\right)$ during the training process, the DCSGAN model incrementally enhances both the quality and classification performance of the generated samples. This iterative refinement allows the generated samples to closely approximate the real data distributions, thereby leveraging the available data to effectively improve overall performance.

**Fig. 5:**
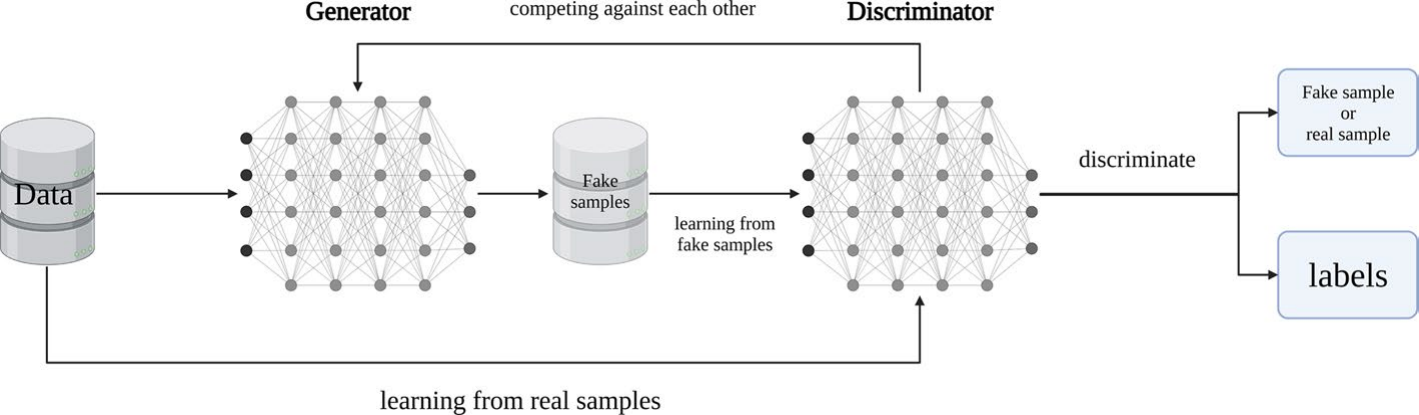
The working principle of DCSGAN

In DCSGAN, the interplay between the Generator and Discriminator contributes to its powerful classification capabilities. With the increased output units in the Discriminator for finer classification, the Discriminator is compelled to learn effective feature representations during the classification task. Through the discrimination between real and generated samples, the Discriminator acquires discriminative feature representations, thereby enhancing the classification performance. Additionally, the Generator's generation of a large number of samples serves as a form of data augmentation and sample expansion, effectively enhancing the diversity and quantity of the original data. This augmentation contributes to improved diabetes classification performance by providing the model with more comprehensive and representative training samples. Figure 5 shows how our proposed model optimizes diabetes classification.

## Result and discussion

### Result of data preprocessing

By counting the number of missing values, the results are shown in Table 5, which shows that Glucose, BloodPressure, SkinThickness, Insulin, and BMI contain missing values, among which SkinThickness and Insulin contain more missing values. The attribute Pregnancies represents the number of pregnancies, and it is reasonable for a value of 0 to exist in the dataset, indicating that some individuals have never been pregnant. Thus, it is considered appropriate and consistent with the nature of the attribute to refrain from filling in missing values for Pregnancies.

**Table 5:** Number of missing values

Features	Number of missing values
Pregnancies	111
Glucose	5
Blood Pressure	35
Skin Thickness	227
Insulin	374
BMI	11
Diabetes Pedigree Function	0
Age	0

**Fig. 6:**
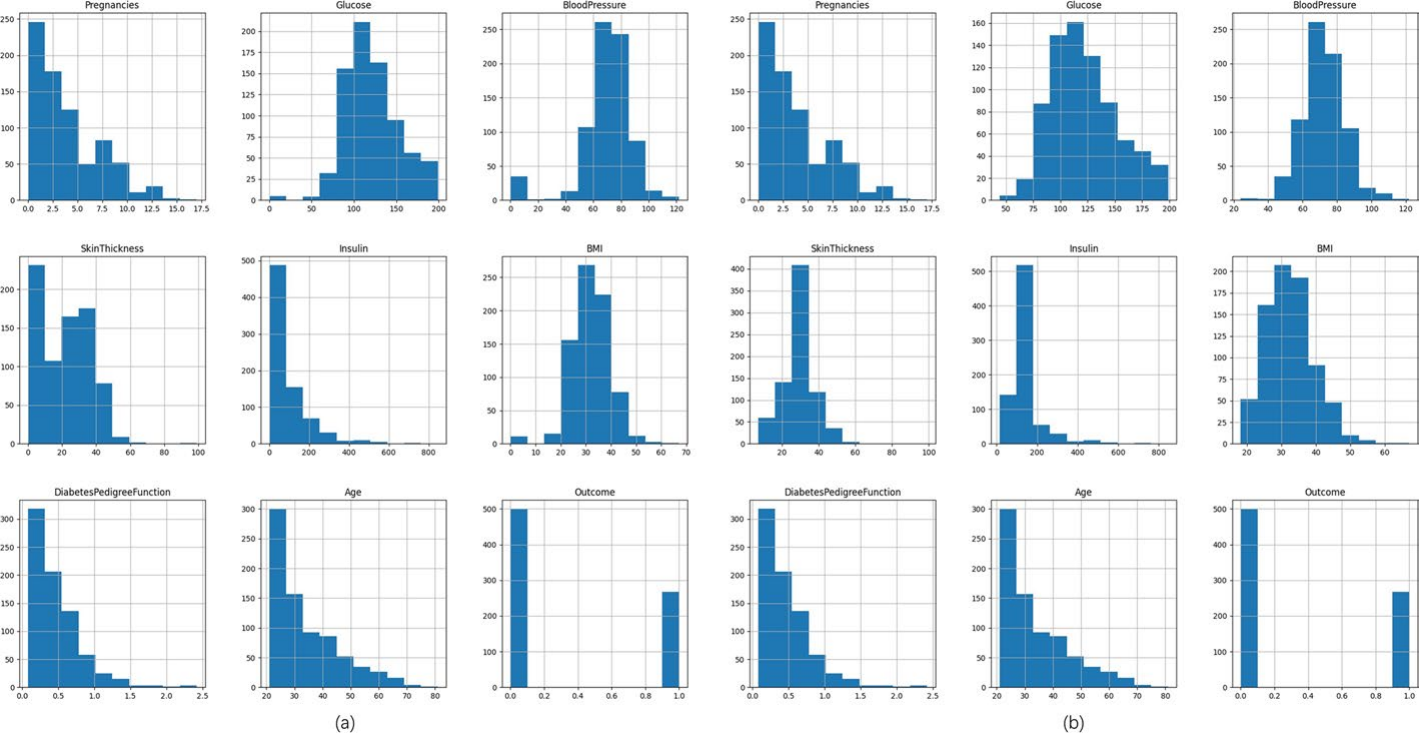
**a** is the data before missing value imputation, **b** is the data after value imputation

After performing the mean median joint filling to handle missing values in the PIMA dataset, the distribution of the dataset is visualized in Fig. 6. The visualization provides insights into the distributions of different attributes. Specifically, it is observed that t after performing missing value imputation on the features Glucose, BloodPressure, SkinThickness, Insulin, and BMI, it is observed that the data distribution of these features tends to align more closely with a normal distribution. This indicates that the imputation process has effectively addressed the missing values, resulting in a more representative and reliable data distribution for these features.

**Fig. 7:**
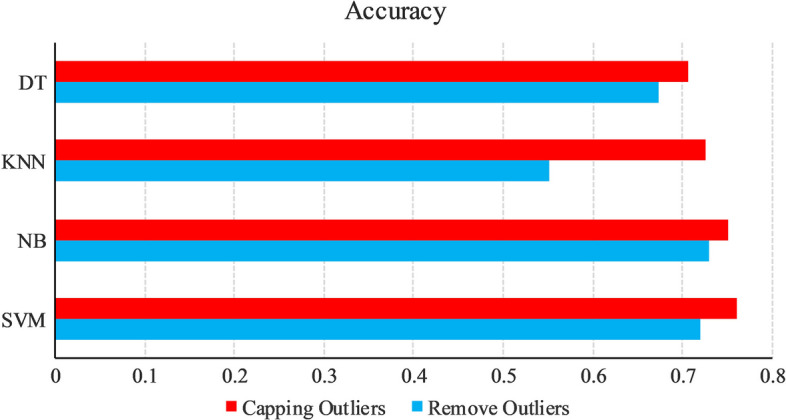
Bar diagram of accuracy comparison

To compare the efficacy of two outlier processing methods, the present study examines the results of utilizing the two methods with four machine learning models SVM, NB, KNN, and DT. Figure 7 illustrates the discernible trends across four models (SVM, NB, DT, KNN), wherein datasets treated with the capping method for outliers exhibited superior accuracy in comparison to datasets with directly removed outliers. Notably, the accuracy of capped datasets consistently surpassed the 70% threshold across all models. Conversely, the accuracy of datasets with directly removed outliers reached or exceeded 70% solely in the NB and SVM models. Based on these results, the present paper employs the capping method for outlier processing.

**Fig. 8:**
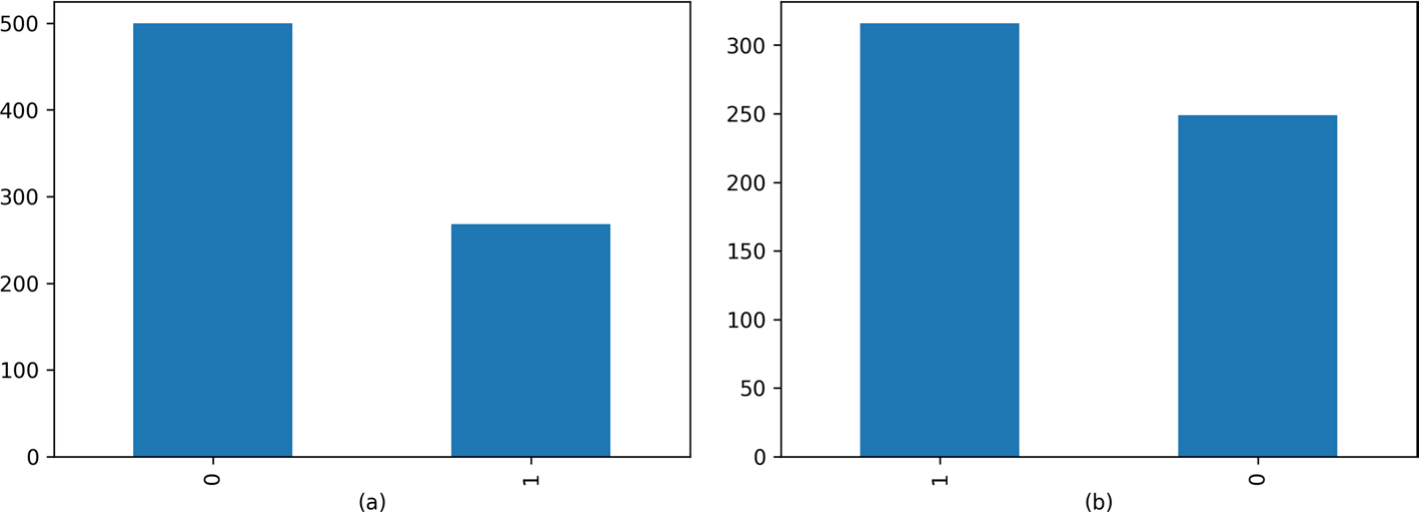
**a** Labels for raw data, **b** Labels after SMOTEENN

To address the issue of sample imbalance in the PIMA dataset, we employed the SMOTEENN hybrid sampling technique. Figure 8 showcases the result obtained after the application of this sampling method.

Figure 8 provides a detailed visualization of the significant improvements achieved by the SMOTEENN algorithm in addressing the issue of sample imbalance. The results presented in Fig. 8 clearly demonstrate a substantial reduction in the disparity of data labels after employing the SMOTEENN algorithm. Initially, the data suffered from a pronounced imbalance, with the "1" labeled samples being only half the number of the "0" labeled samples. However, through the implementation of the SMOTEENN mixed sampling technique, a significant decrease in label frequency variation was observed, effectively alleviating the previously observed data imbalance.

### Result of correlation analysis

**Fig. 9:**
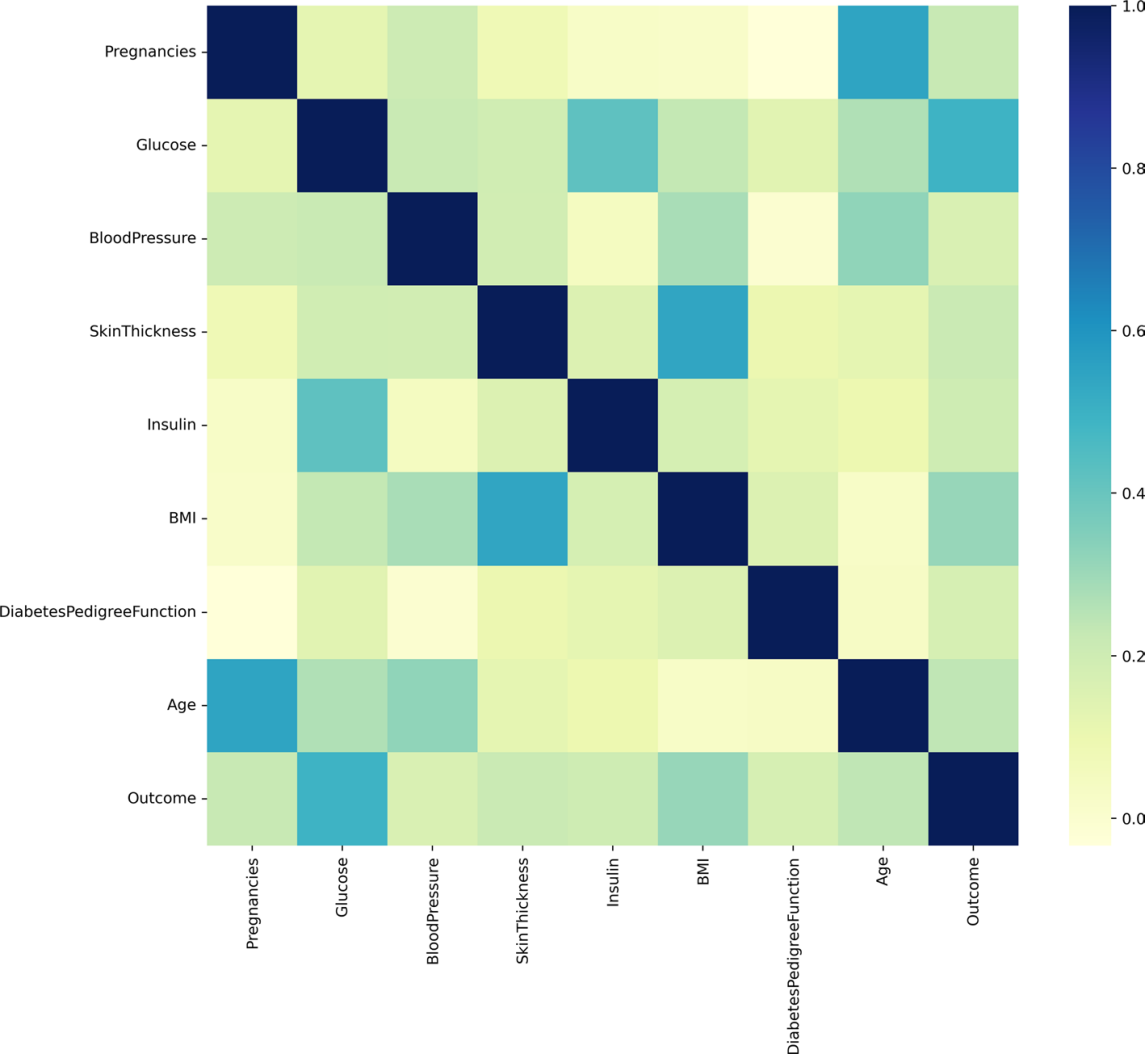
Correlation matrix

Upon exploring the correlation of features in the PIMA dataset, we generated a correlation coefficient heat map as illustrated in Fig. 9. The results indicated that Glucose exhibited a stronger correlation with the outcome compared to other features. To delve deeper into the impact of the features on the outcome, we further utilized logistic regression for conducting a correlation analysis.

Given the limited interpretability of deep learning models, we have employed logistic regression to conduct correlation analysis. This approach enables us to quantify the specific degree of influence that features have on the results. By leveraging logistic regression, we aim to gain deeper insights into the impact of individual features on the outcomes, facilitating further investigations into their influence in subsequent analyses.

Table 6 presents the results of the logistic regression, revealing insightful findings on the relationship between the features and Outcome in the PIMA dataset. The results indicate that Pregnancies and Glucose have a significant effect on Outcome, while BloodPressure, SkinThickness, and Insulin do not. Specifically, for each unit increase in Pregnancies, the probability of Outcome being 0 decreases by 11.767%, and for each unit increase in Glucose, the probability of Outcome being 0 decreases by 3.633%. Similarly, BMI and DiabetesPedigreeFunction also have a significant effect on Outcome, with each unit increase in BMI leading to an 8.867% decrease in the probability of Outcome being 0, and each unit increase in DiabetesPedigreeFunction resulting in a 58.112% decrease in the probability of Outcome being 0. On the other hand, Age does not have a significant effect on Outcome as the significance p-value is 0.175, indicating that the original hypothesis cannot be rejected.

**Table 6:** Result of logistic regression for correlation analysis

	Regression coefficients	Standard error	Wald	*P*	OR	OR 95% confidence interval	
						Upper limit	Lower limit
Pregnancies	− 0.125	0.032	14.953	0.000***	0.882	0.828	0.94
Glucose	− 0.037	0.004	90.025	0.000***	0.964	0.956	0.971
BloodPressure	0.009	0.009	1.021	0.312	1.009	0.992	1.026
SkinThickness	− 0.003	0.013	0.062	0.803	0.997	0.971	1.023
Insulin	0.001	0.002	0.164	0.686	1.001	0.997	1.004
BMI	− 0.093	0.018	27.028	0.000***	0.911	0.88	0.944
DiabetesPedigreeFunction	− 0.87	0.297	8.56	0.003***	0.419	0.234	0.75
Age	− 0.013	0.01	1.838	0.175	0.987	0.969	1.006

Dependent variable: Outcome

***, **, * represent 1%, 5%, 10% significance levels, respectively

### Comparison with other models

Convolutional neural networks, deep neural networks, support vector machines, plain Bayesian, K-nearest neighbor algorithm, and decision trees were compared with our proposed DCSGAN using tenfold cross-validation, a commonly used method for machine learning model evaluation that assesses the generalization ability of the model. The original dataset was divided into 10 disjoint subsets, with one used as the validation dataset and the remaining nine used for training. The model was trained on the nine training datasets and evaluated on the validation dataset, and this process was repeated 10 times using different validation datasets. The final evaluation results were obtained by averaging the 10 evaluations, thus avoiding evaluation errors caused by inappropriate data partitioning.

According to the observations from Table 7, The DSGAN model demonstrated exceptional performance in both binary and tertiary classification tasks, achieving the highest accuracy rates of 96.27% and 99.31% respectively. Furthermore, the model exhibited impressive results across multiple evaluation metrics including precision, F1_score, recall, and AUC. Specifically, the precision, F1_score, recall, and AUC values were observed to be 0.9698, 0.9698, 0.9698, and 0.9702 respectively. These outstanding performance indicators affirm the effectiveness and robustness of the DSGAN model in accurately classifying the given data samples. And a comparative analysis with recent studies was conducted, as presented in Table 8. The findings reveal that our results yielded the highest accuracy rate, demonstrating the superior performance of our approach.

**Table 7:** Result of DCSGAN compare to other models

	Binary accuracy	Trinary accuracy	Precision	F1_score	Recall	Auc
DCSGAN	0.9627	0.9931	0.9698	0.9698	0.9698	0.9702
CNN	0.8271	0.8229	0.6981	0.6930	0.6952	0.6930
DNN	0.7357	0.6095	0.7089	0.7078	0.7084	0.7809
SVM	0.8827	0.7978	0.8893	0.9494	0.9176	0.8984
NB	0.8590	0.8321	0.8749	0.8860	0.8794	0.8607
KNN	0.9501	0.8811	0.9312	0.9778	0.9529	0.9407
DT	0.9224	0.8469	0.9222	0.9468	0.9354	0.9225

**Table 8:** Comparison with state-of-the-art methods

Authors	Models	Classification accuracy (%)
Krishnamoorthi et al. [7]	LR, KNN, SVM, RF	83
Saxena et al. [6]	KNN, RF, DT, MLP	79
Garcia-Ordas et al. [13]	VAE, SAE, CNN	92.31
Bukhari et al. [15]	ABP-SCGNN	93
Gnanadass [18]	NB, LR, RF, AB, GBM, XGB	77.54
Maniruzzaman et al. [10]	LDA, QDA, NB, GPC, SVM, ANN, AB, LR, DT, RF	92.26
Hayashi and Yukita [19]	Re-RX with J 48 graft	83.83
Alneamy et al. [20]	TLBO, FWNN, FLNN, FFWNN	88.67
Chang et al. [21]	NB, RF, J48	79.57
Ours	DCSGAN	96.27

**Fig. 10:**
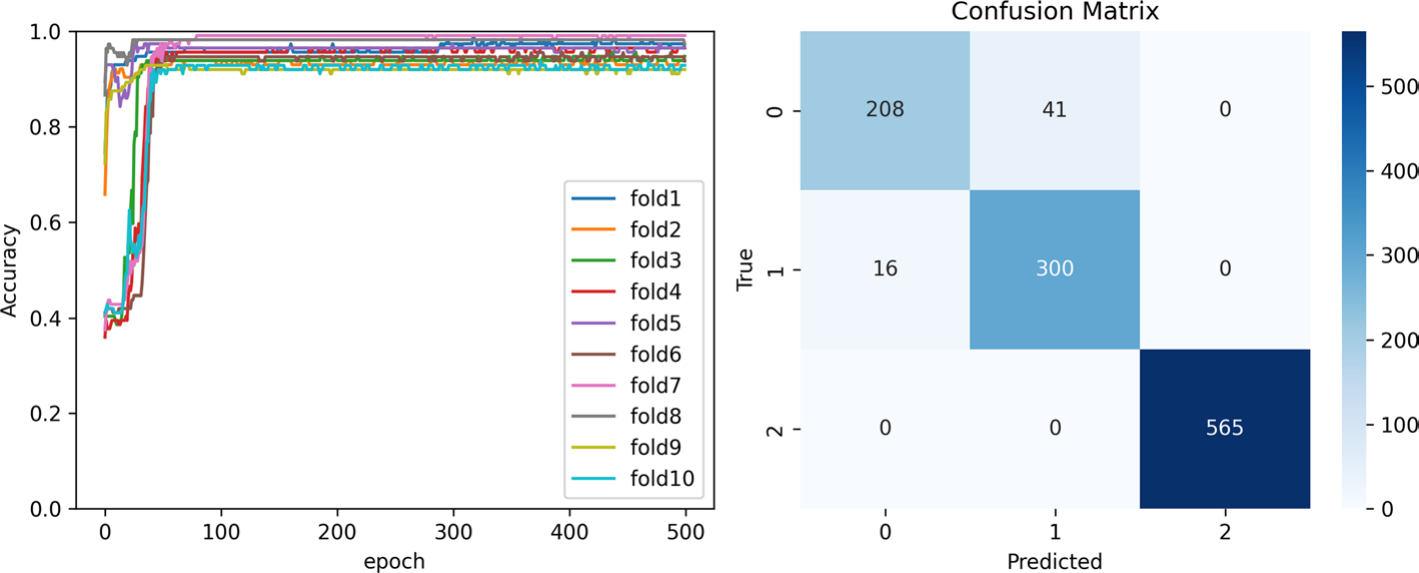
Result of DCSGAN

In Fig. 10, we present a detailed depiction of the training process and the final confusion matrix achieved by the DCSGAN model. The visual representation clearly illustrates the exceptional classification ability demonstrated by our proposed model. The confusion matrix showcases the accurate assignment of samples to their respective classes, underscoring the model's robustness and effectiveness in accurately classifying the dataset. These findings provide compelling evidence of the outstanding performance exhibited by the DCSGAN model in the realm of classification.

### Result on other data set

To assess the generalizability of our model, we validated it on 13 datasets obtained from the GEO database. As shown in Table 9, DCSGAN was found to be inferior only to the Convolutional Neural Network (CNN) and Naive Bayes in the GSE15932 dataset and inferior to the Convolutional Neural Network in the GSE71416 dataset. However, it outperformed other models in terms of accuracy across the remaining 11 datasets.

**Table 9:** Result of GEO dataset

	SVM	NB	DT	NN	CNN	DNN	DCSGAN
GSE76894	0.8157	0.7667	0.7952	0.8524	0.8352	0.8145	**0.9079**
GSE76895	0.6895	0.7290	0.6600	0.7200	0.8239	0.6881	**0.8436**
GSE23343	0.5333	0.6500	0.4500	0.4667	0.6424	0.5870	**0.9992**
GSE161355	0.5429	0.5667	0.7286	0.4524	0.7921	0.4849	**0.8844**
GSE71416	0.7000	0.7000	0.9000	0.7000	**0.9549**	0.7000	0.9179
GSE55650	0.6400	0.7900	0.6400	0.7400	0.8705	0.4783	**0.9491**
GSE55100	0.5400	0.8600	0.7300	0.8200	0.8289	0.5454	**0.9799**
GSE55098	0.5400	0.8600	0.5400	0.8200	0.8289	0.5454	**0.9102**
GSE55099	0.5400	0.7700	0.5999	0.7800	0.8583	0.5460	**0.8657**
GSE15932	0.6714	0.7524	0.6238	0.5429	**0.8451**	0.5313	0.7500
GSE19420	0.7167	0.7167	0.7389	0.5667	0.8451	0.7143	**0.8570**
GSE66738	0.5000	0.5999	0.5071	0.5036	0.6239	0.5263	**0.9548**
GSE25462	0.8000	0.8000	0.8400	0.8400	0.8767	0.6800	**0.8999**

Bold numbers represent the highest accuracy among all the models

## Conclusion

Diabetes, a condition lacking effective treatment, necessitates preventive measures to halt its progression. In this regard, we propose a machine learning-based framework, MOG, for accurate and reliable diabetes diagnosis. The framework integrates essential components, including data preprocessing, SMOTEENN, and classification model development, to achieve precise diagnostic outcomes. To enhance the integrity and quality of the dataset, comprehensive data preprocessing techniques, encompassing missing value imputation and outlier handling using the capping method, are employed. Additionally, by relabeling the PIMA dataset based on glucose levels, effective categorization into three distinct classes—diabetes, prediabetes, and non-diabetes—is achieved. This classification scheme offers valuable insights into the dynamics of disease progression. Furthermore, the imbalance in the PIMA dataset is addressed using the SMOTEENN technique.

The primary contribution of this study lies in the development of the DCSGAN model, which leverages adversarial neural networks for classification tasks. The model exhibits exceptional performance, achieving impressive accuracy rates of 96.27% and 99.31% for dichotomous and trichotomous tasks, respectively. Furthermore, the DCSGAN model demonstrates its ability to generalize well across diverse scenarios by surpassing other models across all 12 datasets in the GEO dataset. Additionally, a logistic regression-based correlation analysis reveals significant biomarkers, including Pregnancies, Glucose, BMI, and Diabetes Pedigree Function, which play a crucial role in diabetes diagnosis. These findings shed light on the factors influencing accurate identification of diabetes cases.

For future research directions, several avenues can be explored. Firstly, the integration of additional biomarkers and clinical variables can be investigated to enhance the accuracy and reliability of diabetes diagnosis. Secondly, extending the MOG framework to encompass longitudinal data analysis and predictive modeling can enable proactive management of diabetes by capturing disease progression patterns over time. Lastly, comprehensive validation and optimization of the proposed framework through extensive clinical trials will ensure its applicability and effectiveness in real-world healthcare settings.

## Data Availability

The data that support the findings of this study are available available on UCI Repository. You can download at Pima Indians Diabetes Database | Kaggle and the datasets shown in Table 2 can download in GEO database by search the appropriate number.

## Abbreviations

SVM: Support vector machines
NB: Naive Bayes
DT: Decision tree
KNN: K nearest neighbors
GAN: Generative adversarial networks
